# Prevalence, Risk Factors, and Microbial Spectrum of Ophthalmia Neonatorum: A Cross-Sectional Study From Central India

**DOI:** 10.7759/cureus.109481

**Published:** 2026-05-23

**Authors:** Pavan Pandey, Shikha Soni, Vibha Pal

**Affiliations:** 1 Community Medicine, Sukh Sagar Medical College, Jabalpur, IND; 2 Microbiology, District Hospital, Dholpur, IND; 3 Ophthalmology, Gajra Raja Medical College, Gwalior, IND

**Keywords:** antenatal care, healthcare acquired conjunctivitis, institutional delivery, maternal genital infections, neonatal conjunctivitis, ophthalmia neonatorum, staphylococcus aureus

## Abstract

Background

Ophthalmia neonatorum (ON) is an acute conjunctival inflammation in neonates that may result in serious complications. Despite advances in intranatal and postnatal care practices, ON remains a significant health concern in several low- and middle-income countries.

Aim

The main aim of this study is to determine the prevalence, associated risk factors, and causative organisms of ON in neonates at a tertiary care hospital in central India.

Methods

A hospital-based, cross-sectional study was conducted over 24 months. Medical records of 2,682 neonates were reviewed; 2,450 neonates met the inclusion criteria. ON was defined clinically by conjunctival redness, eyelid swelling, or purulent discharge within 28 days of birth.

Results

Among the 2,450 neonates whose medical records were included in the study, 74 cases of ON were identified, yielding a prevalence of 3.02%. None of the affected neonates developed serious complications or blindness. The strongest predictors of ON included prolonged rupture of membranes (OR 4.56, p < 0.001), out-of-hospital delivery (OR 6.28, p < 0.001), neonatal intubation (OR 4.78, p < 0.001), and inadequate antenatal visits (fewer than three) (OR 3.08, p < 0.001). Other factors, such as prolonged labour, neonatal resuscitation, and use of orogastric feeding tubes, also showed significant associations. The most frequently isolated organism was *Staphylococcus aureus* (29.7%), followed by *Streptococcus pneumoniae* (16.2%), *Klebsiella pneumoniae* (13.5%), *Escherichia coli* (6.8%), *Pseudomonas aeruginosa* (4.1%), and *Chlamydia trachomatis* (8.1%). About 21.6% of the samples showed no bacterial growth, suggesting either viral causes or self-limiting infections.

Conclusions

ON remains a notable neonatal morbidity in central India. Strengthening antenatal care, promoting institutional deliveries, and enforcing strict infection-control practices during neonatal interventions are essential to reduce the burden of ON.

## Introduction

Ophthalmia neonatorum (ON) remains a significant neonatal health concern in many parts of the world [1]. It is an acute inflammation of the conjunctiva, characterised by redness, discharge, and swelling of the eyelids [1]. If left untreated, ON can lead to corneal ulceration, scarring, perforation, and even permanent blindness [2]. The prevalence and clinical impact of ON vary widely depending on geographical, socio-economic, and healthcare contexts [3]. The aetiology of ON includes chemical, bacterial, and viral agents [3]. Chemical conjunctivitis typically arises from ocular prophylaxis measures like silver nitrate and is often self-limiting [1,4]. Infectious causes, however, are more concerning. Among bacterial pathogens, *Chlamydia trachomatis* and *Neisseria gonorrhoeae* are predominant and are associated with maternal genital infections [3]. Viral causes, such as herpes simplex virus (HSV), add further complexity to ON’s clinical spectrum [1,4].

In low-resource settings, ON prevalence is disproportionately high due to barriers in accessing maternal and neonatal healthcare services [3]. For instance, studies have identified that neonates born outside healthcare facilities or in the absence of skilled birth attendants are at higher risk [3]. Moreover, in regions with weaker health systems, the burden of ON is exacerbated by delayed diagnosis and inadequate treatment. Late presentation and underreporting often obscure the true burden of ON. Risk factors for ON include premature rupture of membranes, prolonged labour, poor maternal hygiene, and inadequate antenatal care [5]. Healthcare facility-related causes of ON include unhygienic delivery environments and insufficient infection control in neonatal care units [5].

This cross-sectional study with retrospective data collection was conducted at Sukh Sagar Medical College, Jabalpur, India, to determine the prevalence, associated factors, and causative organisms of ON.

## Materials and methods

A single-centre, hospital-based, cross-sectional study was conducted at Sukh Sagar Medical College, Jabalpur, Madhya Pradesh, India, and its associated hospital to evaluate the prevalence, risk factors, and causative organisms of ON. The ethical clearance for this study was granted by the Institutional Ethics Committee (IEC) via protocol number SSMC/IEC/2024/CM-06, dated August 27, 2024. A consent waiver was granted by the IEC, as the data were retrospectively collected from prefilled medical records. No data were collected directly from patients or their guardians. There was no face-to-face interaction with any participants. The total duration of the study was 24 months (from July 2022 to June 2024).

The study participants consisted of neonates born at or referred to Sukh Sagar Medical College, Jabalpur, and associated hospitals. The participants included neonates with a confirmed diagnosis of ON and those without ON. The inclusion criteria were as follows: neonates aged 0-28 days; neonates born at or referred to/admitted to the study hospital; and complete medical records available for analysis.

The following were excluded from the study: neonates with incomplete medical records and neonates visiting only the OPD without the need for admission. A whole-population census approach was adopted, wherein all neonates meeting the inclusion criteria during the specified study period were included [6]. A total of 2,682 neonates constituted the study universe.

Data collection procedure

ON was defined as conjunctivitis occurring within the first 28 days of life, characterised by one or more of the following clinical signs [7]: conjunctival redness, eyelid swelling, and/or purulent or mucopurulent discharge from one or both eyes. Diagnosis was confirmed based on documented clinical presentations recorded in neonatal medical records and, where applicable, microbiological identification of causative organisms. The authors and research team thoroughly reviewed hospital records (clinical sheets, laboratory reports, and discharge summaries, etc.) to screen neonates born at or admitted to the hospital during the study period. We used clinical signs, viz., conjunctival redness, eyelid swelling, eye discharge, and other signs indicative of ON, as diagnostic criteria [7]. The study institute has a clinical screening checklist to identify the most common clinical signs, symptoms, and clinical conditions among neonates. The attending paediatric consultant or neonatologist mandatorily completes this checklist for every admitted child. This checklist has a dedicated section for eye examinations to screen neonates for common inherited and acquired ophthalmic disorders. We classified neonates as having ON or not based on the data recorded in the ophthalmic section of the checklist. We excluded neonates from this study if this section was incomplete, and neonates who only visited the OPD due to a lack of detailed medical records.

Data related to potential risk factors were extracted from maternal and neonatal records, including maternal factors such as antenatal care visits, presence of genital infections, mode of delivery (vaginal or caesarean section), place of delivery, condition of liquor, and premature rupture of membranes. Neonatal factors included prematurity, low birth weight, Apgar scores at birth, need for oxygen, intubation, and other interventions. Institutional factors included neonatal interventions (e.g., intubation, catheterisation, orogastric feeding, and need for ventilator support), complications, and treatment details.

Institutional protocol for microbiological examination

Swabs were collected aseptically from the conjunctiva by the staff nurse/paediatrician using sterile cotton-tipped applicators. Separate swabs were used for each eye. Swabs were immediately placed in sterile transport media to maintain pathogen viability. Samples were transported to the microbiology laboratory within an hour of collection to prevent degradation. They were inoculated onto blood agar, chocolate agar, and MacConkey agar plates. Plates were incubated at 37°C for 24-48 hours under appropriate conditions (aerobic or microaerophilic). Growth on culture plates was examined for colony morphology, haemolysis patterns, and pigmentation. Gram staining was performed for preliminary identification. Biochemical tests, such as catalase, coagulase, and oxidase tests, were conducted to confirm bacterial species. In cases where *C. trachomatis* was suspected, PCR-based diagnostic methods were employed. Isolated organisms were subjected to antibiotic susceptibility testing using the Kirby-Bauer disk diffusion method [8]. Results were reported in accordance with Clinical and Laboratory Standards Institute (CLSI) guidelines (institutional licensed printed copy) [9].

The authors developed several standard operating protocols to minimise information bias during data collection. A standardised data extraction tool was developed and strictly adhered to. This tool included detailed guidelines for extracting information uniformly across all medical records, with clearly defined criteria and standardised definitions for all variables to ensure consistency and reduce subjective interpretation. Data from multiple sources within the hospital records, such as neonatal charts, microbiological reports, discharge summaries, and nursing notes, were cross-referenced. All personnel (nursing and medical students) involved in data extraction underwent training to ensure familiarity with the protocol and uniform interpretation of clinical variables. In addition, medical records of all neonates identified as having ON were double-checked and cross-verified with laboratory reports by the authors. Statistical analysis was undertaken using Stata 17.0 (Stata license: single-user, perpetual, serial number: 401706383980; StataCorp LLC, College Station, TX, USA) [10]. Descriptive statistics were used to determine the prevalence and distribution of ON, while inferential statistics (Chi-square or Fisher’s exact test for categorical risk factors and independent t-test or Mann-Whitney U test for continuous variables) assessed associations between risk factors and outcomes. A p-value of <0.05 was considered statistically significant.

## Results

During the study period, a total of 2,682 neonates were born at or admitted to the hospital. A total of 232 neonates were excluded from analysis: 160 neonates had an incomplete or empty ophthalmic section of the checklist, and 72 neonates did not have essential information related to antenatal or peripartum events (e.g., mode and place of delivery). The remaining 2,450 neonates were included.

The study included a total of 2,450 neonates, among whom 74 (3.02%) cases of ON were identified. None of the participants developed major complications, blindness, or recurrence. The characteristics of the participants are shown in Table 1.

**Table 1:** Characteristics of Participants (n = 2450)

Variable	n	%
Age (days)		
0-7	1158	47.3%
8-14	712	29.1%
15-21	354	14.4%
22-28	226	9.2%
Gender		
Male	1285	52.4%
Female	1165	47.6%
Gestational Age		
Preterm (<37 weeks)	438	17.9%
Term (≥37 weeks)	2012	82.1%
Birthweight		
Low (<2.5 kg)	572	23.3%
Normal (≥2.5 kg)	1878	76.7%

Among the 74 cases of ON, 16 cases (21.6%) showed no bacterial growth. The most frequently isolated organism was *Staphylococcus aureus* (22 cases, 29.7%), followed by *Streptococcus pneumoniae* (12 cases, 16.2%). Gram-negative bacteria such as *Klebsiella pneumoniae* and *Escherichia coli* were identified in 10 cases (13.5%) and five cases (6.8%), respectively. Additionally, *Pseudomonas aeruginosa* was isolated in three cases (4.1%). Chlamydial infections were also detected in six cases (8.1%). There were no cases of mixed infections involving two or more bacterial species.

Table 2 shows the results of univariable logistic regression to identify the determinants of ON among study participants.

**Table 2:** Risk Factors for ON Among Study Participants ON: Ophthalmia Neonatorum

Risk Factor	ON Cases (n = 74)	Non-ON Cases (n = 2376)	Odds Ratio	95% CI	p-value
Maternal Factor					
Prolonged rupture of membranes (PROM >18 hours)	32 (43.2%)	340 (14.3%)	4.56	2.84-7.33	<0.0001
Place of delivery outside hospital (home or during transportation)	28 (37.8%)	210 (8.8%)	6.28	3.84-10.26	<0.0001
Maternal genital infections	15 (20.3%)	160 (6.7%)	3.52	1.95-6.35	0.0002
< 3 ANC visits	42 (56.8%)	710 (29.9%)	3.08	1.93-4.92	<0.0001
Long duration of labour (>24 hours)	18 (24.3%)	320 (13.5%)	2.07	1.20-3.56	0.0149
Low socioeconomic status	42 (56.8%)	1250 (52.6%)	1.17	0.74-1.83	0.5158
Caesarean section	32 (43.2%)	1095 (46.1%)	0.89	0.55-1.43	0.6815
Maternal complications leading to prolonged hospital stay	28 (37.8%)	465 (19.6%)	2.52	1.55-4.09	0.0003
Maternal Hb (mean ± SD)	10.2 ± 1.1 g/dL	10.3 ± 1.2 g/dL	0.89	0.63-1.37	0.332
Maternal age (mean ± SD)	26.4 ± 4.8 years	26.8 ± 4.9 years	1.09	0.68-1.48	0.450
Neonatal Factors					
Neonatal intubation	19 (25.7%)	160 (6.7%)	4.78	2.77-8.26	<0.0001
Preterm birth (<37 weeks)	22 (29.7%)	416 (17.5%)	2.00	1.19-3.36	0.0089
Multiple pregnancy	4 (5.4%)	110 (4.6%)	1.18	0.40-3.41	0.7686
APGAR @ 5 minutes < 6	12 (16.2%)	250 (10.5%)	1.65	0.86-3.17	0.1600
Neonatal resuscitation	22 (29.7%)	450 (18.9%)	1.81	1.09-3.01	0.0241
Orogastric feeding tube	30 (40.5%)	650 (27.4%)	1.81	1.13-2.90	0.0142
Antenatal corticosteroids (ANC) steroids	8 (10.8%)	180 (7.6%)	1.48	0.69-3.18	0.3295
Venous catheterisation	15 (20.3%)	350 (14.7%)	1.47	0.83-2.62	0.1956
Phototherapy for neonatal jaundice	10 (13.5%)	300 (12.6%)	1.08	0.55-2.13	0.859

## Discussion

The present study found that the prevalence of ON was 3.02%. Studies from India have reported varying prevalence of ON, reflecting differences in period, methodology, and population characteristics. A hospital-based study conducted in New Delhi in 2016 reported that ON was prevalent in 4.1% of neonates born at their institute [5]. Wadhwani et al. (2011, Delhi) [11] reported a prevalence of 4%, while Shah et al. (2017, Gujarat) reported ON in 35% of neonates [12]. A more recent study from Kerala, Mammooty et al. (2021), found a prevalence of 3.5%, despite the implementation of an azithromycin prophylaxis programme [13]. Earlier microbiological studies, such as Mohile et al. (2002, Delhi), noted ON prevalence of around 7.2% [14].

In comparison with these studies, the prevalence of 3.02% appears moderate and may reflect gradual improvements in antenatal coverage, infection-control measures, and institutional delivery practices in India. However, for several reasons, this estimate (3%) is unlikely to represent the true burden of ON in India. First, the present study was undertaken in a private tertiary care hospital, whereas the majority of deliveries in India take place in public hospitals [15]. The quality of care, particularly with regard to infection control, is typically more robust in private hospitals than in public hospitals [16-19]. Moreover, women who deliver in private hospitals are generally better off economically, have higher levels of literacy, and receive better antenatal care compared with those delivering in public hospitals [15]. These differences in maternal demographic characteristics may have contributed to the lower prevalence of ON among neonates born in private healthcare settings. Taken together, these considerations strongly suggest that the actual burden of ON in central India may be higher than that observed in this study. Thus, these findings should be interpreted with caution, as they may obscure the true prevalence of ON, delay the prioritisation of preventive strategies, and ultimately hinder efforts to further reduce avoidable blindness and improve neonatal outcomes [20].

A higher ON prevalence has been reported from neighbouring South Asian countries. Gul et al. (2010, Pakistan) reported an incidence of 6.5%, attributing the burden to unhygienic delivery practices and limited prophylaxis [1]. Studies from Iran, such as Amini et al. (2008) [21] and Khoshdel et al. (2012) [22], documented ON prevalence ranging between 8% and 12%, often linked to maternal genital tract infections and perinatal risk factors. A systematic review reported that the pooled prevalence and incidence of ON were 7.79% and 2.04%, respectively [3].

Taken together, these comparisons demonstrate that the prevalence of ON in the present study represents an intermediate burden - lower than that in many low-resource countries but higher than that in high-income countries. The findings underscore the continuing relevance of ON as a neonatal morbidity in India, where antenatal care is inadequate, home deliveries persist, or maternal genital infections remain untreated [15].

Our study identified several determinants associated with ON. Most of these risk factors are consistent with evidence from India, neighbouring South Asian countries, and global literature. Among our participants, prolonged rupture of membranes (PROM) increased the odds of ON nearly fivefold. Wadhwani et al. (2011, Delhi) also reported PROM as a key determinant [11]. Studies from Ghana (prevalence 12.5%) [23] and Malawi (prevalence 11.4%) [24] highlighted PROM as one of the strongest maternal risk factors for ON [24].

Our study found that out-of-hospital delivery increased ON risk more than sixfold, and this finding is similar to several other studies. In a study conducted in Kerala, the prevalence of ON was higher among those born outside institutional settings [13]. Gul et al. (2010) also reported non-institutional deliveries as a strong determinant of ON [1]. Similarly, a Kenyan study reported higher ON prevalence in home-delivered neonates [25].

Preterm birth was independently associated with ON in our study. This is consistent with findings from Gujarat, where low birth weight and prematurity were key contributors [12]. Internationally, a study conducted in Qatar also reported higher ON incidence among preterm infants requiring respiratory support [26]. Neonatal intervention (intubation and orogastric tubes) was also significantly associated with ON in our study. This is consistent with reports of healthcare-associated conjunctivitis (HAC) from Indian neonatal intensive care units (NICUs) [5,11] and global HAC studies, where device-related exposures remain major determinants [3].

Our study did not find any association with caesarean section, socioeconomic status, maternal age, multiple pregnancy, APGAR score <6, or maternal anaemia.

In the present study, the predominant causative organisms isolated from conjunctival swabs were *S. aureus* and *Klebsiella*, followed by other Gram-negative bacilli. Multiple Indian studies corroborate our findings. Shah et al. (2017, Gujarat) reported *S. aureus* in 46% of cases and *P. aeruginosa* in 23% [12]. Goel et al. (2016, New Delhi) reported that among the 20 culture-positive cases of ON, the most common pathogens were *E. coli* (35%), *S. aureus* (25%), *Klebsiella* spp. (15%), and *P. aeruginosa* (10%) [5]. Wadhwani et al. (2011, Delhi) found coagulase-negative staphylococci as the leading isolates (60%), with *S. aureus* and *E. coli* also identified [11]. Earlier work by Mohile et al. (2002, Delhi) reported *S. epidermidis* (57%) and *C. trachomatis* (24%) as the major isolates [14]. More recently, a study from Kerala (2021) also found *S. aureus* among the causative organisms [13]. These data demonstrate that although the microbial profile varies by geography, staphylococci and Gram-negative bacilli remain the most consistent pathogens in Indian neonates.

Neighbouring countries also report a predominance of Gram-positive cocci and enteric Gram-negative bacilli. In Pakistan, Gul et al. (2010) found *S. aureus* and *Pseudomonas* as leading agents [1]. Shrestha et al. (2021) from Nepal reported that the most common isolated organisms from ON cases were coagulase-negative staphylococci (40%), *S. aureus* (30%), *Klebsiella *(20%), and *Pseudomonas* (10%) [27]. Iranian studies highlighted diverse microbial causes, including *Chlamydia* and *Staphylococcus* species [21,22]. These findings, in line with ours, show that while classical sexually transmitted pathogens (*N. gonorrhoeae* and *C. trachomatis*) remain relevant, staphylococci and Gram-negative bacilli are increasingly important causes of ON in South Asia and the Middle East.

Taken together, this microbial shift has important implications. First, prophylactic regimens effective only against *N. gonorrhoeae* may have limited impact in current contexts. Second, the rising role of hospital-acquired and resistant strains, such as methicillin-resistant *S. aureus* (MRSA), reported in Indian NICUs, highlights the importance of infection-control measures and antimicrobial stewardship. Lastly, surveillance of local microbial trends should guide empirical treatment policies to reduce complications and recurrence.

The present study has a few limitations. First, it was a single-centre study conducted in a private tertiary care hospital, which may limit the generalisability of findings to public healthcare settings and the wider population. Second, data collection relied on the accuracy and completeness of medical records, creating potential information bias. Third, neonates with incomplete records were excluded, which may have introduced selection bias. Finally, viral causes of ON were not systematically investigated, and some culture-negative cases may therefore have remained undiagnosed.

## Conclusions

This hospital-based cross-sectional study from central India found a prevalence of ON of 3.02%. The burden, though lower than that reported in many regional and global studies, indicates that ON remains an important neonatal morbidity. Determinants significantly associated with ON included PROMs, prolonged labour, inadequate antenatal care, delivery outside hospitals, preterm birth, and invasive neonatal procedures such as intubation and orogastric tube use. The microbial spectrum was dominated by *S. aureus* and Gram-negative bacilli such as *Klebsiella*, consistent with findings from other Indian and regional studies. Our findings highlight the need to strengthen antenatal care, ensure institutional deliveries, promote timely treatment of maternal genital infections, and enforce strict infection-control practices in NICUs.
